# The Future of Natural Killer Cell Immunotherapy for B Cell Non-Hodgkin Lymphoma (B Cell NHL)

**DOI:** 10.1007/s11864-021-00932-2

**Published:** 2022-03-08

**Authors:** Yaya Chu, Margaret Lamb, Mitchell S. Cairo, Dean A. Lee

**Affiliations:** 1 Department of Pediatrics, New York Medical College, Valhalla, NY USA; 2 Hematology, Oncology, and Blood and Marrow Transplant Section, Nationwide Children’s Hospital, Columbus, OH USA; 3 Department of Pediatrics, College of Medicine, The Ohio State University, Columbus, OH USA; 4 Department of Medicine, New York Medical College, Valhalla, NY USA; 5 Department of Pathology, New York Medical College, Valhalla, NY USA; 6 Department of Microbiology and Immunology, New York Medical College, Valhalla, NY USA; 7 Department of Cell Biology and Anatomy, New York Medical College, Valhalla, NY USA; 8 Center for Childhood Cancer and Blood Disorders, Abigail Wexner Research Institute at Nationwide Children’s Hospital, Columbus, OH USA

**Keywords:** NK cells, Antibody-dependent direct cytotoxicity, Non-Hodgkin lymphoma, Chimeric antigen receptor

## Abstract

Natural killer (NK) cells have played a critical—if largely unrecognized or ignored—role in the treatment of B cell non-Hodgkin lymphoma (NHL) since the introduction of CD20-directed immunotherapy with rituximab as a cornerstone of therapy over 25 years ago. Engagement with NK cells leading to lysis of NHL targets through antibody-dependent cellular cytotoxicity (ADCC) is a critical component of rituximab’s mechanism of action. Despite this important role, the only aspect of B cell NHL therapy that has been adopted as standard therapy that even indirectly augments or restores NK cell function is the introduction of obinutuzumab, a CD20 antibody with enhanced ability to engage with NK cells. However, over the last 5 years, adoptive immunotherapy with effector lymphocytes of B cell NHL has experienced tremendous growth, with five different CAR T cell products now licensed by the FDA, four of which target CD19 and have approved indications for some subtype of B cell NHL—axicabtagene ciloleucel, brexucabtagene autoleucel, lisocabtagene maraleucel, and tisagenlecleucel. These T cell-based immunotherapies essentially mimic the recognition, activation pathway, and cytotoxic machinery of a CD19 antibody engaging NK cells and lymphoma targets. Despite their efficacy, these T cell-based immunotherapies have been difficult to implement because they require 4–6 weeks of manufacture, are costly, and have significant toxicities. This renewed interest in the potential of cellular immunity—and the manufacturing, supply chain, and administration logistics that have been addressed with these new agents—have ignited a new wave of enthusiasm for NK cell-directed therapies in NHL. With high safety profiles and proven anti-lymphoma efficacy, one or more new NK cell-directed modalities are certain to be introduced into the standard toolbox of NHL therapy within the next few years, be it function-enhancing cytokine muteins, multi-domain NK cell engagers, or adoptive therapy with expanded or genetically modified NK cells.

## Introduction

Approximately 90,000 new cases of lymphoma are diagnosed in the USA per year, approximately 90% of which (estimated 81,560 in 2021 per American Cancer Society) are non-Hodgkin lymphoma (NHL), and the majority of these are B cell NHL [1–3]. While the survival rate for newly diagnosed children and adolescents with B cell NHL treated with chemotherapy and antibody-based regimens has more than doubled from the 1970s to the early 2000s (45% to > 90%) [1], the prognosis is dismal in patients with relapsed/refractory B cell NHL [4, 5, 6••, 7, 8, 9•]. Novel approaches with CD19- and BCMA-targeted CAR T cellular immunotherapy have recently been approved by the FDA for patients with relapsed/refractory B cell NHL including CD19 CAR T cells [10, 11••, 12••, 13••]. However, treatment with CAR T cells is complicated by high cost, manufacturing logistics, and toxicity.

In contrast, NK cells have similar cytotoxic effector mechanisms as T cells but appear to have a broader safety profile and are more amenable to generating allogeneic ready-to-infuse (a.k.a. “off-the-shelf”) products. Like T cells, NK cells can also be genetically modified for antigen-specific targeting [3]. Liu et al. recently reported the safety and efficacy of cord blood derived CD19 CAR NK cells in patients with relapsed/refractory CD19 B-cell NHL and chronic lymphocytic leukemia (CLL) [13^••^]. We now summarize the clinical and preclinical experience with NK cells and CAR NK cells in B-cell NHL.

## Endogenous NK cells in B-cell NHL

**Fig. 1:**
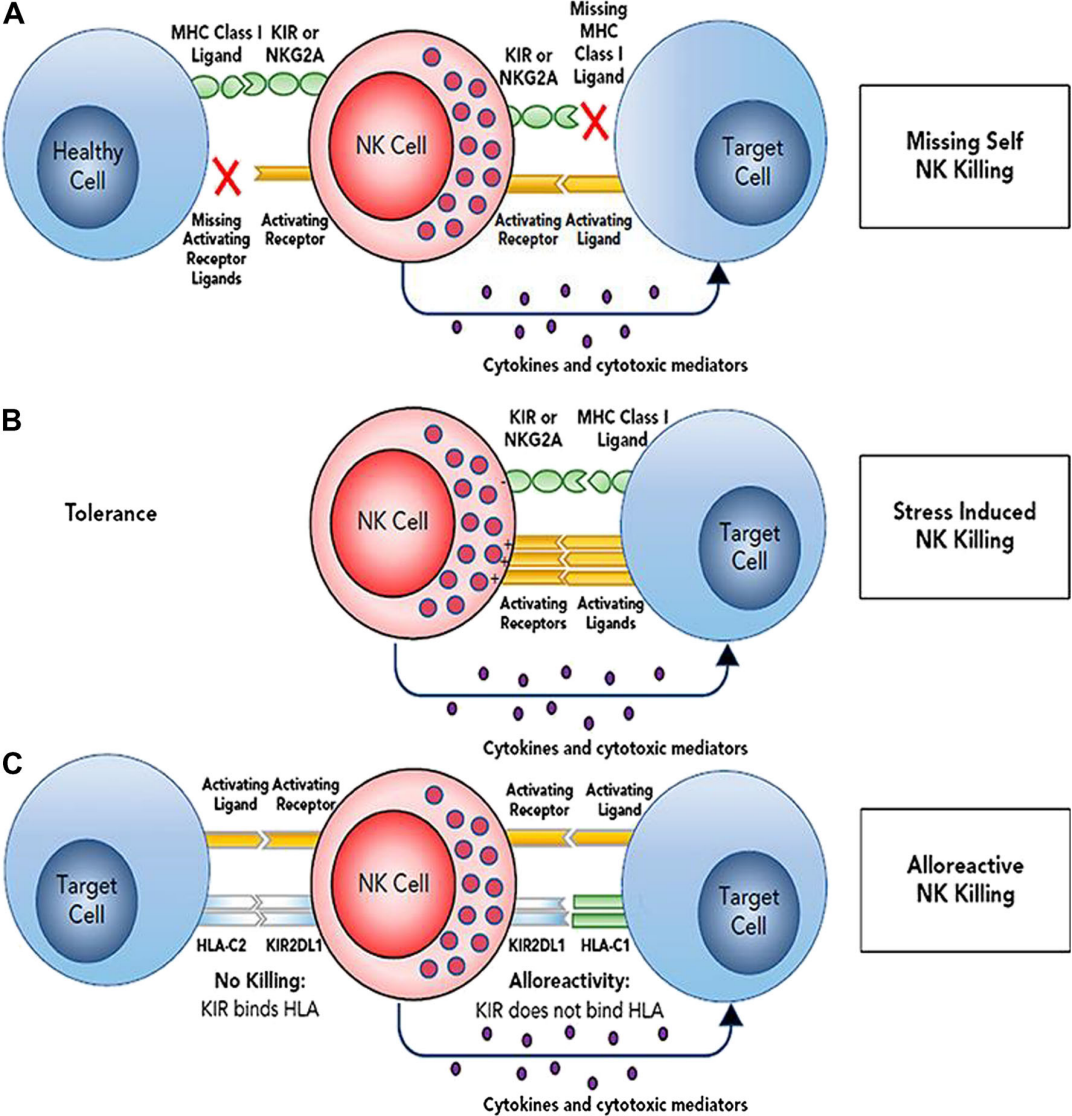
NK cell recognition of target cells. NK cell effector function is dependent on a balance of activating and inhibitory signals to distinguish between healthy cells (tolerance) and cancer or virally infected cells. The absence of MHC Class I ligand (missing self, observed with HLA downregulation or induced with KIR-ligand mismatch in HLA mismatched recipient/donor pairs) combined with upregulation of stress-induced activating receptor ligands leads to target recognition, NK cell activation, and cytotoxicity. Reproduced with permission from: Cooley S, Parham P, Miller JS. Strategies to activate NK cells to prevent relapse and induce remission following hematopoietic stem cell transplantation. Blood. 2018;131(10):1053-62. doi: 10.1182/blood-2017-08-752170.

NK cells are innate lymphocytes that play a key role in the recognition of cells that are cancerous or virus-infected. NK cell activation or inhibition (tolerance) is determined by an integrated balance of signals from NK cell activating and inhibitory receptors binding to their corresponding ligands on target cells [14] (Fig. 1). As part of our first line of defense, NK cells exert their effector function directly via cellular cytotoxicity and indirectly via proinflammatory cytokine secretion. In the last few decades, NK cells have moved to the forefront of immune oncology for several reasons. First, improved understanding of NK cell biology, discovery of new NK cell sources, and advances in NK cell culture techniques have made it possible to expand and activate NK cells for adoptive cell therapy. In addition, in contrast to T cells, NK cells do not depend on antigen presentation but instead utilize a balance of inhibitory and activating cell receptors that recognize self and stress ligands to determine effector function. Without the need for antigen presentation, NK cells can be ubiquitously effective even in cancers where tumor specific antigens remain elusive. Finally, allogeneic NK cell therapy is safe with no reports of dose limiting toxicities including graft versus host disease (GVHD), even with minimal HLA matching [15–17].

In patients with lymphoma, decreased number and function of NK cells portends a poor prognosis [18–20, 21•]. Methods of immune evasion in the lymphoma tumor microenvironment (TME) include immune checkpoints, hypoxia-induced immune modulation, and aberrant NK cell receptor/ligand expression [22–27]. Similar to the well described graft-versus tumor effect in hematopoietic stem cell transplant (HSCT) for leukemia, early and robust NK cell recovery after autologous and allogeneic HSCT in lymphoma is associated with improved survival [28–32]. To facilitate improved NK cell function after autologous HSCT in lymphoma, several studies administered low dose recombinant IL-2 as maintenance immunotherapy to prevent relapse [33–35]. In a study of relapsed/refractory NHL patients undergoing autologous HSCT, 10 patients received low dose recombinant IL-2 (rIL2) as maintenance therapy for 12 months after autologous HSCT. Following rIL2 therapy, peripheral blood (PB) samples from patients had a significant increase in NK cell number, function, and CD16-mediated ADCC compared to their baseline number and function before therapy [33]. None of the 10 patients treated on the protocol had relapse of their disease, with a median follow up of 16 months from the start of rIL2. Interestingly, two patients who still had residual disease after HSCT showed complete resolution of the residual disease while receiving rIL2 therapy. A similar study by Miller et al. using low dose IL-2 after autologous HSCT in patients with lymphoma and breast cancer also demonstrated a more than 10-fold increase in PB NK cells with enhanced cytotoxicity against resistant cell lines [35]. To build off of these early studies, clinical trials utilizing adoptive transfer of autologous ex vivo-activated NK cells or lymphokine-activated killer cells in patients with lymphoma emerged with only modest activity [34, 36–40] (Table 1). In addition, although feasible, the use of autologous NK cells was costly, often required more than one apheresis procedure, and NK cell doses were limited to around 10^7^/kg [44]. To improve clinical efficacy, more recent studies have utilized highly functional expanded NK cells and/or allogeneic NK cell sources to facilitate the graft versus tumor effect. Yang et al. demonstrated the safety of expanded random healthy donor PB NK cells for treatment of malignant lymphoma and other solid tumors [42]. Multiple doses of up to 3 × 10^7^/kg were administered with no dose limiting toxicities and no GVHD. Clinical responses, however, were limited with only 8/17 patients with stable disease as best response overall response and no complete responses. Although the safe use of donor derived NK cells represented progress in the field, alloreactive NK cells alone did not appear to be enough to eliminate bulk disease.

**Table 1:** Published results of adoptive NK cell therapy for lymphoma

Lymphoma type	NK Cell product	NK Cell source	NK cell dose	Lymphodepleting chemotherapy	Combination therapy	Outcomes	Author	Year
HL (*n* = 3), NHL (*n* = 8), Breast Cancer (*n* = 1)	Expanded	Autologous	6.8 × 10^8^–4 × 10^10^ NK Cells for 1 dose	None	IL-2	Not Reported	Lister et al. [36]	1995
B cell NHL (*n* = 6)	Overnight IL-2 Activation	Haploidentical	2 × 10^6^–40 × 10^6^/kg for 1 dose	Fludarabine, Cyclophosphamide	IL-2 + rituximab	2 CR, 2 PR	Bachanova et al. [41]	2010
NHL (*n* = 6) HL (*n* = 2) MM (5)	Overnight IL-2 Activation	Haploidentical	1 × 10^5^–2 × 10^7^/kg for 1 dose	None		Primary endpoint safety, 8/13 in remission	Klingemann et al. [40]	2013
NHL (*n* = 2) and Advanced Solid Tumors (*n* = 18)	Expanded	Unrelated healthy donor	1 × 10^6^–3 × 10^7^/kg for 1–3 doses	None		8/17 SD	Yang et al. [42]	2016
NHL (*n* = 15)	Overnight IL-2 Activation	Haploidentical	0.5–3.27 × 10^7^/kg for 1 dose	Fludarabine, Cyclophosphamide, Methylprednisolone	IL-2 + rituximab	4/15 ORR, 2/15 CR	Bachanova et al. [21]	2018
NHL (*n* = 9)	Expanded	Autologous	1 × 10^6^–1 × 10^7^/kg for 1 dose	none	rituximab	7/9 with CR	Tanaka et al. [43]	2020

*NK* natural killer, *HL* Hodgkin lymphoma, *NHL* non-Hodgkin lymphoma, *IL*-*2* interleukin-2, *CR* complete response, *PR* partial response, *MM* multiple myeloma

## Targeting NK cells to NHL with antibodies

Antibody-based therapy has become a critical part of the treatment landscape in hematologic malignancies in the past few decades and several monoclonal antibodies have been FDA approved that target the lymphoma-specific antigens CD19 (loncastuximab tesirine and tafasitimab-cxix), CD20 (rituximab, obinutuzumab, ofatumumab, ibritumomab tiuxetan), CD30 (brentuximab vedotin), CD52 (alemtuzumab), CD38 (daratumumab, isatuximab), CD79b (polatuzumab vedotin), and CCR4 (mogamulizumab). One of the mechanisms by which antibodies mediate tumor cell lysis is through ADCC by NK cells. NK cells recognize the Fc portion of antibodies bound to the surface of target cells via the Fc-gamma receptor III (CD16). Optimal efficacy of antibody therapy depends on high number and function of NK cells. In patients with DLBCL and follicular lymphoma treated with anti-CD20 monoclonal antibodies, low pre-treatment NK cell count was associated with shorter progression free survival and decreased overall survival compared to patients with higher pre-treatment NK cells [45]. NK cell ADCC has been exploited in antibody therapy by systemic cytokine stimulation of endogenous NK cells [46–50] or in combination with adoptive NK cell therapy [43, 51]. Autologous cytokine-expanded NK cells were combined with chemotherapy and rituximab in 9 patients with relapsed CD20-positive lymphoma patients to enhance ADCC [43]. A single dose of escalating expanded NK cells (1 × 10^6^/kg, 3 × 10^6^/kg, and 10 × 10^6^/kg) was given on the day after rituximab. Complete responses were observed in 7/9 patients and there was a significant increase in PB NK cells and cytolytic activity in all patients two weeks after infusion. In a phase II trial, patients with relapsed/refractory CD20^+^ NHL were given IL-2-activated haploidentical PB NK cells after lymphodepleting chemotherapy [21^•^]. A single dose of 0.5–3.27 × 10^7^ NK cells/kg was given in combination with IL-2 every other day × 6 doses and weekly rituximab × 4 doses. The NK cells were well tolerated and elicited responses in 4/14 evaluable patients, including 2 complete responses. Importantly, the authors noted improved NK cell persistence and effector function in patients with higher endogenous IL-15 at the time of NK cell infusion highlighting the importance of in vivo cytokine stimulation. Based on this and similar observations, subsequent studies utilizing IL-15 alone or in combination with adoptive NK cell infusion are being investigated. ALT-803 is an IL-15 super agonist complex developed to mimic physiologic trans-presentation of IL-15 and prolong the half-life. A phase I study of ALT-803 in 33 adult patients with hematologic malignancies who relapsed after allogeneic HSCT demonstrated that the cytokine therapy was safe and significantly increased NK and CD8^+^ T cell number and function [52]. Although only 4 patients had objective responses (1 CR, 1 PR, 2 SD) after 5 doses of ALT-803, the enhanced immune milieu and tolerability has led to further trials of ALT-803 and related compounds combined with rituximab [53] and/or adoptive NK cell therapy, including in lymphoma (NCT02890758) (Table 2).

**Table 2:** Clinical trials utilizing adoptive NK cell therapy for lymphoma

NK Cell Source	Combination Therapy	Disease	Phase	NCT Number	Status	Country
Umbilical Cord Blood NK Cells	AFM13	CD30+ HL and NHL	Phase I	NCT04074746	Recruiting	USA
Haploidentical NK Cell Enriched DLI	Haploidentical HSCT	NHL, HL, MM, CLL	Phase I	NCT03524235	Recruiting	USA
Umbilical Cord Blood NK Cells	rituximab, Autologous HSCT	NHL	Phase II	NCT03019640	Recruiting	USA
Universal Donor Expanded NK Cells	ALT803	HL, NHL, AML, MDS, ALL, CML, CLL, Solid Tumors	Phase I	NCT02890758	Recruiting	USA
Natural Killer Cells (Source Unspecified)	rituximab	B Cell Lymphoma	Phase I/II	NCT02843061	Completed	China
Umbilical Cord Blood NK Cells	Umbilical cord blood HSCT +/- rituximab	HL, NHL AML, ALL, MDS, CML	Phase II	NCT02727803	Recruiting	USA
Haploidentical NK Cells	Autologous HSCT	Lymphoma, Neuroblastoma, Solid Tumors	Phase I	NCT02130869	Completed	USA
Haploidentical NK Cell Enriched DLI	Haploidentical HSCT	Lymphoma, AML, ALL, MDS, Neuroblastoma, Rhabdomyosarcoma	Phase I/II	NCT01386619	Completed	Germany, Switzerland
Donor Derived Expanded NK Cells	Allogeneic HSCT	Lymphoma, Leukemia	Phase I	NCT01287104	Completed	USA
NK-92 Cell Line		NHL, HL, Leukemia, Myeloma	Phase I	NCT00990717	Completed	Canada
Donor Derived NK Cells	Allogeneic HSCT	Lymphoma, Leukemia, MM, MDS, Brain Tumors, Soid Tumors	Phase I/II	NCT00823524	Completed	Korea
Donor Derived NK Cells	Allogeneic HSCT	NHL, HL, ALL, AML, MDS, CLL, CML, Myeloma	Phase I/II	NCT00789776	Completed	USA
Haploidentical NK Cells	Lymphodepleting chemotherapy and IL-2	NHL, ALL, AML, MDS, CML, JMML	Phase I	NCT00697671	Completed	USA
Haploidentical NK Cells	Autologous HSCT	Lymphoma, Leukemia, Myeloma	Phase I	NCT00660166	Completed	USA
Haploidentical NK Cells	Lymphodepleting chemotherapy and IL-2	T Cell LLy, ALL, AML, JMML, MDS	Phase I	NCT00640796	Completed	USA
Haploidentical NK Cell Enriched DLI	Haploidentical HSCT	Lymphoma	Phase I	NCT00586703	Completed	USA
Matched Family Donor NK Cell Enriched DLI	Matched Family Donor HSCT	Lymphoma	Phase I	NCT00586690	Completed	USA
Donor Derived NK or T Cells	Allogeneic HSCT with alemtuzumab	Lymphoma, Leukemia	Phase II	NCT00536978	Completed	USA
Donor Derived NK Cells	Allogeneic HSCT, GM-CSF, rituximab	CD20 + NHL, CLL	Phase I	NCT00383994	Completed	USA
Donor Derived NK Cells	Lymphodepleting chemotherapy, rituximab, IL-2	CD20 + NHL, CLL	Phase I/II	NCT00625729	Terminated	USA
Umbilical Cord Blood NK cells	Lymphodepleting chemotherapy, Lenalidomide, rituximab	Leukemia, Lymphoma	Phase I	NCT02280525	Active, Not Recruiting	USA
Umbilical Cord Blood NK cells	Umbilical cord blood HSCT +/− rituximab	HL, NHL, ALL, AML, MDS, CML, CLL, Myeloma	Phase I	NCT01619761	Active, Not Recruiting	USA

*NK* natural killer, *NCT* national clinical trial, *HSCT* hematopoietic stem cell transplant, *HL* Hodgkin lymphoma, *NHL* non-Hodgkin lymphoma, *AML* acute myelogenous leukemia, *MDS* myelodysplastic syndrome, *ALL* acute lymphoblastic leukemia, *CML* chronic myelogenous leukemia, *LLy* lymphoblastic lymphoma, *JMML* juvenile myelomonocytic leukemia, *IL*-*2* interleukin-2, *CR* complete response, *PR* partial response, *MM* multiple myeloma, *CLL* chronic lymphocytic leukemia, *DLI* donor lymphocyte infusion, *GM*-*CSF* granulocyte-monocyte colony-stimulating factor

## Targeting NK cells to NHL with CARs

**Fig. 2:**
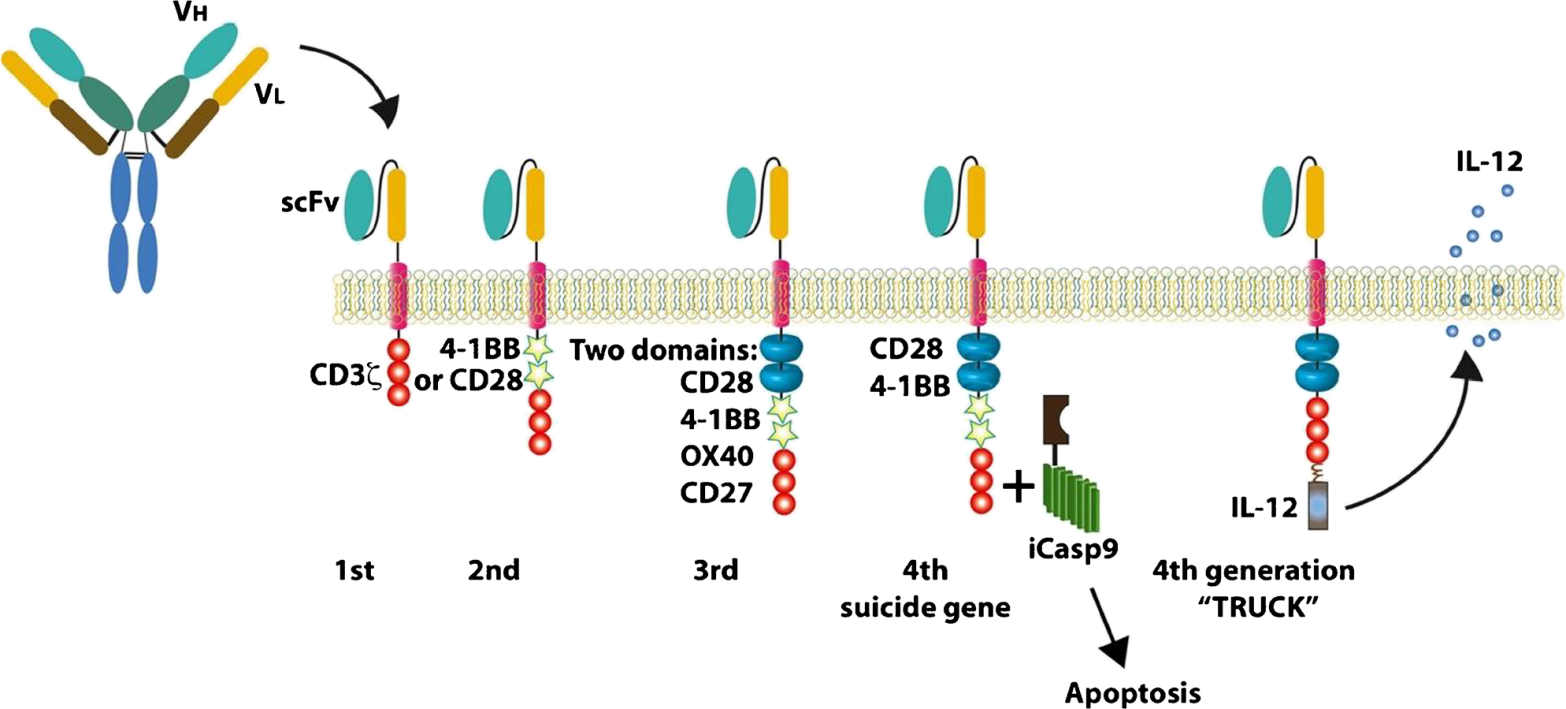
Chimeric antigen receptor (CAR) structure. CAR constructs typically consist of an extracellular antigen binding domain (single chain variable fragment (scFv)), a transmembrane domain, and an intracellular signaling domain comprised of a stimulatory domain without (first generation) or with one (second generation) or more (third generation) costimulatory domains. Fourth generation CARs include additional elements such as cytokine secretion or inducible suicide genes. Reproduced with permission from: Barth MJ, Chu Y, Hanley PJ, Cairo MS. Immunotherapeutic approaches for the treatment of childhood, adolescent and young adult non-Hodgkin lymphoma. Br J Haematol. 2016;173(4):597-616. doi: 10.1111/bjh.14078.

**Fig. 3:**
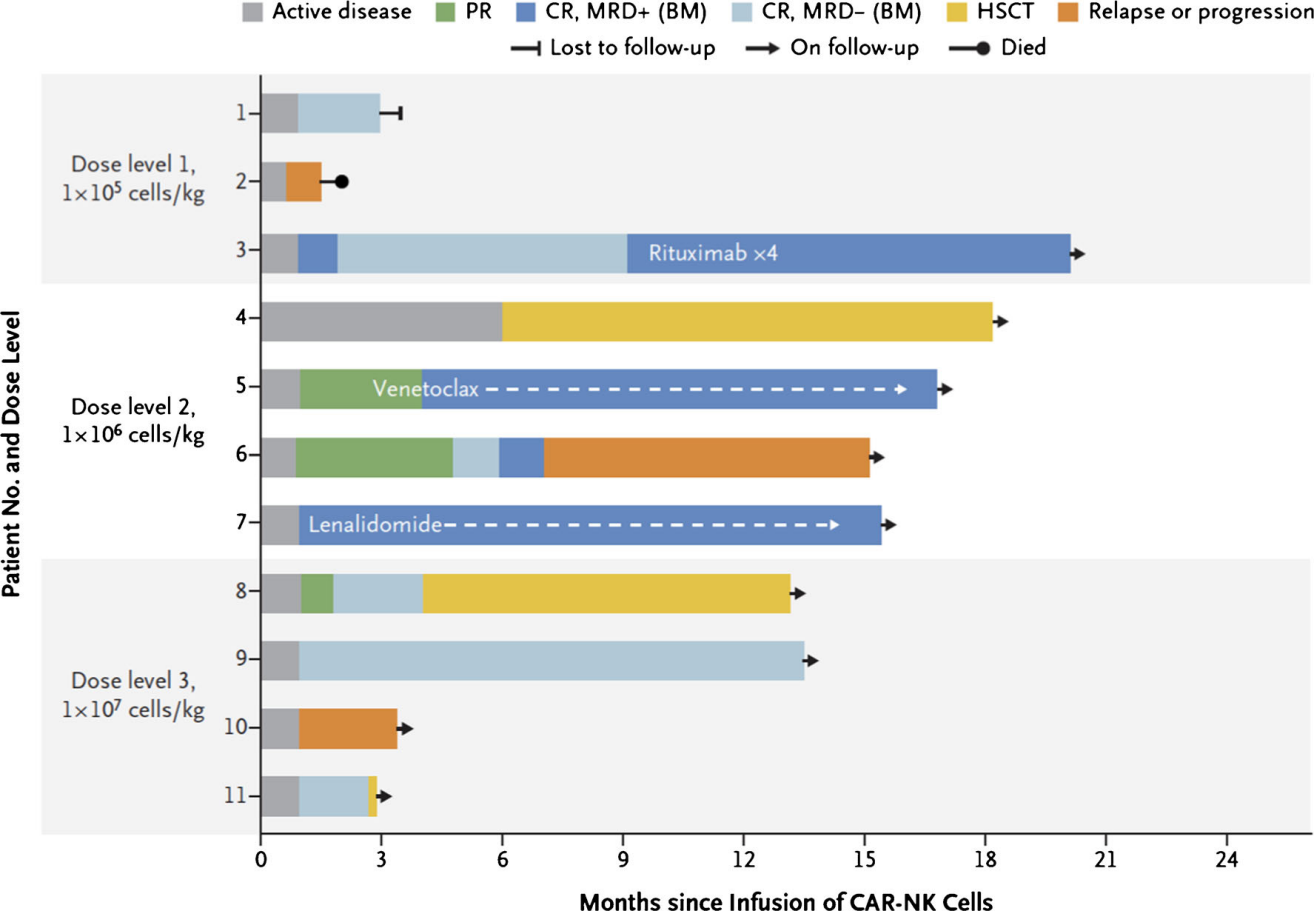
Clinical responses to CD19 CAR-NK therapy for CD19 positive lymphoid malignancies. Clinical outcomes of 11 patients treated with cord blood derived, IL-15 expressing, CD19 CAR NK Cell with an inducible caspase 9 suicide gene (iC9/CAR.19/IL-15 CB NK cells). The legend denotes partial response (PR), complete response (CR), minimal residual disease (MRD), and hematopoietic stem cell transplantation (HSCT). Reproduced with permission from: Liu E, Marin D, Banerjee P, Macapinlac HA, Thompson P, Basar R, et al. Use of CAR-Transduced Natural Killer Cells in CD19-Positive Lymphoid Tumors. N Engl J Med. 2020;382(6):545-53. doi: 10.1056/NEJMoa1910607.

With the success of CD19 CAR T cell therapy in hematologic malignancies, there has been a push to develop CAR NK cells targeting a wide variety of tumor antigens. Currently, the FDA-approved CAR T cell products are manufactured from autologous T cells due to the risk of GVHD from the native T cell receptor in allogeneic CAR T cells. Manufacturing CAR T cells on a patient-by-patient basis is costly, time consuming, and often fails due to poor T cell function in heavily pretreated cancer patients. CARs usually contain a single chain variable fragment from a monoclonal antibody, a transmembrane hinge region, a signaling domain such as CD3-zeta, and one or more co-stimulatory domains such as CD28, 4-1BB, or 2B4 (CD244) [54, 55] (Fig. 2). Since NK cells utilize many of the same signaling domains and activation pathways as T cells, NK cells can also be redirected to specifically target tumor antigens by the introduction of a CAR, which then permanently recapitulates the targeting function of an antibody and activation pathways for cytotoxicity. Moreover, the addition of a CAR does not abrogate the NK cell’s innate recognition of cancer through endogenous receptors. One clear advantage of NK cells is the ability to use donor derived, “off-the-shelf” NK cells that minimize or eliminate the need for HLA matching without the risk of GVHD. Recently, Liu et al. developed a novel cord blood derived, IL-15 expressing, CD19 CAR NK cells with an inducible caspase 9 suicide gene (iC9/CAR.19/IL-15 CB NK cells) [56]. In a phase I/II trial, 11 patients with relapsed/refractory CD19-positive cancers were given escalating doses of these CD19 CAR NK cells after lymphodepleting chemotherapy with fludarabine and cyclophosphamide [13^••^]. Of 11 patients treated (5 with CLL, 4 with NHL), 8 patients (73%) had an objective response, including 7 patients with a complete response (Fig. 3). The NK cell infusions were safe with no CRS, neurologic toxicity, or GVHD, and the NK cells were detectable (by assaying for the vector transgene) for at least 12 months after infusion. Progress in the field of genetic modification of NK cells has opened the door for true “off-the-shelf” CAR NK cell therapy. Additional CAR NK cell targets for lymphoma currently under clinical investigation include CD19, CD22, and CD7 (Table 3).

**Table 3:** CAR-NK clinical trials for lymphoma

Target Antigen	Tumor Type	Type of NK Cell	Phase	NCT Number	Status	Country
CD19	NHL	Not Listed	Early Phase I	NCT04639739	Not yet recruiting	China
CD19	ALL, CLL, Mantle Cell and Follicular Lymphoma	NK-92	Phase I/II	NCT02892695	Unknown	China
CD19	ALL, NHL, CLL	Cord Blood	Phase I	NCT04796675	Recruiting	China
CD19	ALL, NHL, CLL	Cord Blood	Phase I/II	NCT03056339	Recruiting	USA
CD19	ALL, CLL, B Cell Lymphoma	Cord Blood	Phase I	NCT04796688	Recruiting	China
CD19	B cell lymphoma, Mantle cell and Follicular Lymphoma	Cord Blood	Phase I/II	NCT03579927	Withdrawn	USA
CD19	B Cell Lymphoma, CLL	iPSC	Phase I	NCT04245722	Recruiting	USA
CD19	B Cell Lymphoma	Not Listed	Early Phase I	NCT03690310	Not yet recruiting	China
CD19/CD22	B cell Lymphoma	Not Listed	Early Phase I	NCT03824964	Unknown	China
CD22	B Cell Lymphoma	Not Listed	Early Phase I	NCT03692767	Not yet recruiting	China
CD7	AML, T-ALL, T-LLy, NK/T-LLy	NK-92	Phase I/II	NCT02742727	Unknown	China

*CAR* chimeric antigen receptor, *NK* natural killer, *NCT* national clinical trial, *NHL* non-Hodgkin lymphoma, *AML* acute myelogenous leukemia, *ALL* acute lymphoblastic leukemia, *LLy* lymphoblastic lymphoma, *CLL* chronic lymphocytic leukemia

## Sources of NK or CAR NK cells for adoptive NK cell-based immunotherapy for B cell NHL

There are numerous donor sources for isolation, purifying, and targeted NK cells for adoptive NK cell-based immunotherapy. These include autologous NK cells from patients, allogeneic NK cells from peripheral blood, umbilical cord blood, CD34 hematopoietic stem cells, embryonic stem cells, and induced pluripotent stem cells, and leukemia- or lymphoma-derived NK cell lines [34, 57–69]. An extensive review of the pros, cons, and experience to date with these NK cell sources has recently been published [70].

## Ex vivo NK cell expansion for adoptive NK cell-based immunotherapy for B cell NHL

### Ex vivo NK expansion with feeder cells for B cell NHL

**Fig. 4:**
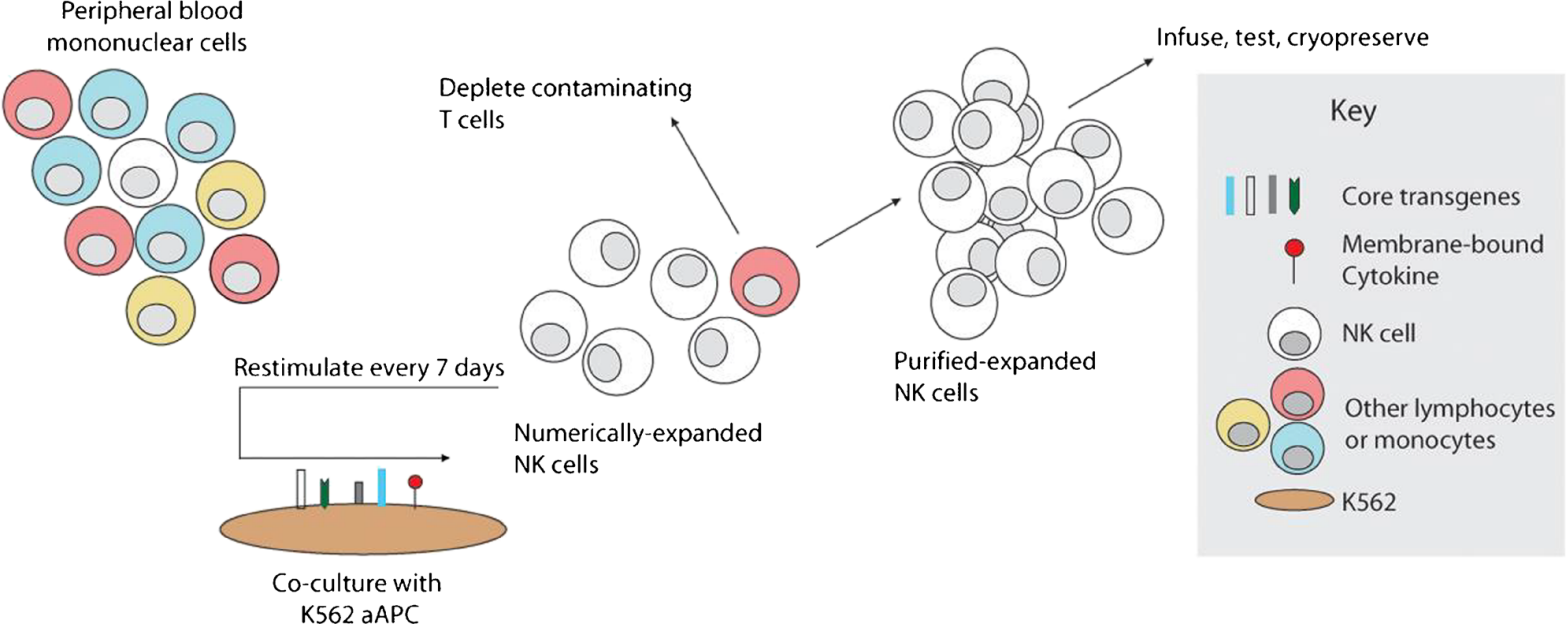
Schema for NK cell manufacturing with genetically-engineered feeder cells. Feeder cells were produced by genetic modification of K562 to express costimulatory molecules and membrane-bound cytokines. To expand NK cells *ex vivo*, unfractionated PBMC are stimulated weekly with irradiated PBMC, inducing rapid proliferation of NK cells and a variable degree of non-specific expansion of T cells. Contaminating T cells may be depleted before or during expansion, and the remaining purified NK cells may be stimulated weekly by the artificial antigen-presenting cells as needed to obtain sufficient numbers. Expanded NK cells may be used directly or cryopreserved for future use. Reproduced with permission from: Denman CJ, Senyukov VV, Somanchi SS et al. (2012), Membrane-Bound IL-21 promotes sustained *ex vivo* proliferation of human Natural Killer cells. PLoS ONE 7(1): e30264. doi: 10.1371/journal.pone.0030264.

To overcome the limitation of small number of active NK cells in the donor PB, NK cells can be ex vivo activated and expanded with feeder cells such as irradiated PBMCs [71–73], Epstein-Barr virus-transformed lymphoblastoid cell lines (EBV-LCL) [74], or gene-modified cell lines such as K562 [75–78]. Preclinical studies demonstrated that highly cytotoxic NK cells can be expanded with irradiated and activated autologous PBMCs to efficiently kill lymphoma cells in vitro and in mice xenografted with human lymphoma cells [71–73]. These NK cells expanded with irradiated autologous PBMC are suitable for allogeneic transfer without the risk of graft-versus-host disease induction [71–73]. Our group and others have successfully expanded active PB NK cells or CB NK cells in vitro by co-culture with irradiated EBV-LCL, or with K562 cells expressing transfected cell-membrane bound IL-15 and 4-1BBL to kill leukemia and B-NHL [74–77]. Lee and colleagues have developed a novel feeder cell line what was engineered to express membrane bound IL-21 and 4-1BB ligand (K562-mbIL21-41BBL) to expand PB NK or CB NK ex-vivo [78]. This method resulted in over 35,000-fold increase in NK cells in 3 weeks while avoiding telomere shortening and NK cell senescence and significant increase in NK cell functional activation against lymphoma (Fig. 4) [78].

### Ex-vivo NK cell expansion without feeder cells for B cell NHL

NK cells can also be ex vivo activated and expanded with feeder-free systems. Clinical responses were observed in 4 of 6 B cell NHL patients who were administered rituximab and haploidentical donor PB NK cells activated with IL2 [41]. However, due to host Treg proliferation stimulated by the IL2, the donor NK cell expansion was inhibited in the peripheral blood [41]. Nicotinamide (NAM) is a form of Vitamin B3. Preclinical studies showed that NAM enhanced expansion (60-80 fold) of functional donor NK cells in feeder-free cultures stimulated with IL-2 and IL-15 for 2 weeks and the expanded NK cells with NAM displayed the increased in vitro cytotoxicity against Burkitt lymphoma (BL) cells, in vivo homing, and survival in immunodeficient mice [79]. Additionally, NK cells can be expanded with a feeder-free, particle-based approach, which uses plasma membrane particles (PM-particles) derived from K562-mbIL15-41BBL or K562-mbIL21-4-1BBL cell lines [80, 81], but preclinical studies in B cell NHL have not yet been reported.

## Preclinical studies of CAR engineered NK cells for B cell NHL

CAR NK therapy is promising and may provide some advantages over CAR T cells such as low risk of on-target/off-tumor toxicity to normal tissues, reduced risk for GVHD, and reduced frequency and severity of cytokine release syndrome (CRS) or immune effector cell-associated neurotoxicity (ICANS) [55]. NK cells can be engineered to express CAR through several technologies such as transposons, messenger ribonucleic acid (mRNA)-mediated gene delivery, lentiviruses, and CRISPR/AAV targeted gene insertion [82]. We and others have engineered expanded PB NK (exPBNK) cells with anti-CD19 CAR or anti-CD20 CAR utilizing retroviral integration or CAR mRNA electroporation to target B cell malignancies including B-NHL [83, 84]. We previously reported that anti-CD20 CAR exPBNK cells had significantly enhanced in vitro cytotoxicity against BL, limit BL tumor metastasis, and extended the survival of NSG mice xenografted with human BL cells [84]. The combination of anti-CD20 CAR exPBNK cells with a histone deacetylase inhibitor romidepsin, which enhanced expression of NKG2D ligands on the surface of BL, significantly killed BL, reduced tumor burden, and extended the survival in NSG mice xenografted with human BL cells [85]. Similarly, engineering the NK-like lymphoma cell line NK92 with an anti-CD19 CAR significantly increased in vitro cytotoxicity and prominently induced apoptosis in rituximab- and obinutuzumab-resistant cell lines and patient-derived cells, and delayed tumor growth in B-NHL xenografts [86]. Liu et al. genetically modified NK cells expanded from cord blood (CB) cells with a retroviral vector (iC9/CAR.19/IL-15) [56]. Their preclinical studies demonstrated that iC9/CAR.19/IL-15 CB NK cells efficiently killed CD19^+^ primary leukemia cells and Raji in vitro and significantly prolonged the survival in a xenograft Raji lymphoma murine model [56]. Emerging preclinical evidence shows that IL-12 and IL-18 plus IL-15 can induce murine and human NK cells with long-lasting memory-like functionality [87]. These cytokine-induced memory-like (CIML) NK cells display increased proliferative capacity, prolonged persistence in vivo, and superior functionality in killing of rituximab-coating Raji lymphoma cells compared with control NK cells in vitro, and substantially reduced growth of established lymphoma tumors in mice [88–90]. Anti-CD19 CAR-modified CIML NK cells displayed significantly increased interferon-γ (IFNγ) secretion and cytotoxicity against NK-resistant lymphoma lines and primary lymphoma tumor cells, and significantly reduced lymphoma burden and significantly improved the survival in human lymphoma xenograft models [91]. Goodridge et al. developed a CAR NK product FT596, derived from a master induced pleuripotent stem cell (iPSC) line engineered to uniformly express anti-CD19-CAR, an enhanced functioning high-affinity, non-cleavable CD16, and a recombinant fusion of IL-15 and IL-15 receptor alpha (IL-15RF) [92]. FT596 showed significantly enhanced clearance of Raji tumor cells in combination with rituximab in a Raji xenografted mouse model [92]. Furthermore, utilizing an allogenic human CD34 engrafted NSG mouse model, FT596 demonstrated improved survival and safety over primary CAR19 T cells, either as a monotherapy or as a combination therapy with rituximab against Raji tumor cells [92]. These preclinical findings have provided the preliminary results to conduct a Phase I dose-finding study of FT596 as monotherapy and in combination with rituximab or obinutuzumab in subjects with relapsed/refractory B cell lymphoma or CLL (NCT04245722).

## Preclinical studies of combinatorial therapies of NK cells for B cell NHL

A large number of preclinical studies of combination therapies incorporating NK cells for B cell NHL have been reported (Table 4), a number of which are summarized below.

**Table 4:** Preclinical studies of human NK therapy for lymphoma

NK source	Activation and expansion method	Genetically engineered	Engineering method	Combination	Lymphoma subtype	Study stage	Year	Ref
NK-92	IL-2	No		N/A	Human BL	Preclinical	1994	[59]
haNK	N/A	Yes (CD16-158V and erIL2)	Transfection/insertion	rituximab	Human CD20+ lymphoma	Preclinical	2016	[60]
CB	Anti-CD3, IL2, IL7, IL12	No	No	N/A	Human BL	Preclinical	2009	[64]
hESC	NK differentiation medium (IL15, IL3, IL7, SCF, Flt3L) and irradiated AFT-24 cells	No	No	rituximab	Human BL	Preclinical	2005	[67]
iPSC	N/A	Yes (ADAM17 knockdown)	CRISPR/Cas9	rituximab	Human BL	Preclinical	2020	[69]
PB	IL12, IL15, IL18	No	No	rituximab	Human BL	Preclinical	2017	[88–90]
PB	NAM, IL2, IL15	No	No	No	Human BL	Preclinical	2011	[79]
PB	irradiated autologous PBMCs	No	No	No	Human BL	Preclinical	2013, 2013, 2017	[71–73]
PB	irradiated k562-mbIL15-41BBL	No	No	No	Human BL	Preclinical	2013	[77]
CB	irradiated k562-mbIL15-41BBL	No	No	No	Human BL DLBCL	Preclinical	2017	[76]
PB	irradiated k562-mbIL21-41BBL	No	No	No	Human BL	Preclinical	2012	[78]
PB	irradiated k562-mbIL15-41BBL	Yes (anti-CD19 CAR)	mRNA electroporation	No	Human BL	Preclinical	2012	[83]
PB	irradiated k562-mbIL15-41BBL	Yes (anti-CD20 CAR)	mRNA electroporation	No	rituximab sensitive and resistant human BL	Preclinical	2015	[84]
PB	irradiated k562-mbIL15-41BBL	Yes (anti-CD20 CAR)	mRNA electroporation	romidepsin	rituximab sensitive and resistant human BL	Preclinical	2017	[85]
NK-92	IL2	Yes (anti-CD19 CAR)	Lentiviral transduction	No	Human BL, DLBCL	Preclinical	2020	[86]
CB	irradiated k562-mbIL21-41BBL	Yes (anti-CD19 CAR-IL15-iC9)	Retroviral transdduction	No	Human BL	Preclinical	2019	[56]
PB	IL12, IL15, IL18	Yes (anti-CD19 CAR)	N/A	No	Human BL	Preclinical	2020	[91]
iPSC (FT596)	N/A	Yes (anti-CD19 CAR-hnCD16-IL15RF)	N/A	rituximab	Human BL	Preclinical	2019	[92]
PB	irradiated k562-mbIL15-41BBL	No	No	Obinutuzumab	Human BL	Preclinical	2015	[93]
PB	No	No	No	CD19/CD16 BiKE CD19/CD22/CD16 TriKE	Human BL	Preclinical	2012	[94•]
PB	No	No	No	161519 TriKE	Human BL	Preclinical	2012	[95]
PB	No	No	No	CD30/CD16A (AFM13) tandem diabody	Human HL	Preclinical	2014	[96]
PB	No	No	No	rituximab	Human BL FL	Preclinical	2016	[97]
PB	No	No	No	N-820	Human BL	Preclinical	2016	[98]
PB	irradiated k562-mbIL21-41BBL	No	No	N-820	rituximab sensitive and resistant human BL	Preclinical	2020	[99]

*CB* cord blood; *PB* peripheral blood; *hESC* human embryonic stem cell; *SCF* stem cell factor; *Flt3L* Flt3 ligand; *iPSC* induced pluripotent stem cell; *ML* memory-like; *NAM* nicotinamide; *iC9* inducible caspase-9; *CAR* chimeric antigen receptor; *hnCD16* high-affinity, non-cleavable CD16; *BL* Burkitt lymphoma; *FL* follicular lymphoma; *mRNA* messenger ribonucleic acid; *Ref*. references

### Combinatorial therapy of NK cells with a novel type II anti-CD20 antibody Obinutuzumab

Obinutuzumab is a humanized, type II anti-CD20 monoclonal antibody glycoengineered to enhance Fc receptor affinity and has been approved for the treatment of patients with previously untreated advanced-stage follicular lymphoma [100, 101]. Our preclinical studies demonstrated that obinutuzmab has significantly enhanced ADCC compared to rituximab and induced apoptosis in BL in vitro, and the combination of obinutuzumab with exPBNK significantly enhanced overall survival of NSG mice xenografted with Raji tumor cells as compared to the combination of rituximab with exPBNK [93]. Our group is currently conducting a clinical trial to evaluate the safety and response rate of obinutuzumab as a single agent alone and in combination with ifosfamide, carboplatin and etoposide (O-ICE) without exogenous NK cells in children, adolescents and young adults with recurrent refractory CD20^+^ mature B-NHL including BL (NCT02393157).

### Combinatorial therapy of NK cells with bispecific or trispecific killer engagers

Bispecific killer engagers (BiKEs) or trispecific killer engagers (TriKEs) are designed to have one “arm” binding to CD16 on NK cells and the other one or two “arms” targeting to the specific antigen(s) on the tumor cells [102]. The engager substitutes for traditional antibody-Fc interactions in mediating the immunological synapse between tumor cells and NK cells to stimulate NK activation and killing [102]. Gleason and colleagues developed an anti-CD16/CD19 BiKE and an anti-CD16/CD19/CD22 TriKE, and showed that they trigger NK cell activation through direct signaling of CD16 to secrete lytic granules and induce BL tumor death via a caspase-3 apoptosis pathway [94•]. A TriKE designated 161519 was developed combining the cytokine IL-15 with the anti-CD16 scFv and the anti-CD19 scFv (98) in order to link cytokine signaling with antigen-specific NK cell activation. Preclinical studies demonstrated that this novel 161519 TriKE induced more degranulation and IFNγ production in NK cells, resulting in the highest level of Raji cell death as compared with rituximab, 1619 BiKE, or controls [95], indicating the potential immunotherapeutic value of 161519 TriKE in B cell NHL.

### Combinatorial therapy of NK cells with IL15 superagonist (N-803) and rituximab

IL-15 shares similar functions with IL-2 but has a distinct advantage over IL-2 for cancer immunotherapy due to its minimal binding to the low-affinity IL-2 receptor CD25, resulting in a lack of effect on Tregs [103]. N-803 is an IL-15 superagonist (originally named ALT-803) that consists of a high-affinity interleukin-15 mutein (IL-15N72D) and a dimeric IL-15 receptor alpha (IL-15Rα)/Fc fusion protein [104]. N-803 has at least 25 times the activity of the wild type IL-15 in vivo and a significantly longer serum half-life in vivo than wild-type IL-15 (25 h vs. < 40 min) [104, 105]. The study from Rosario et al. showed that the combination of N-803 with rituximab significantly increased the expression of granzyme B and perforin, IFNγ production, and ADCC of human NK cells against BL cell lines or primary follicular lymphoma cells [97]. The study supports the future clinical investigation of N-803 plus NK cells and anti-CD20 mAbs in patients with aggressive B cell NHL.

### Combinatorial therapy of NK cells with N-820, a novel antibody-N-803 fusion

N-820 was generated by fusing four single-chains of rituximab to the N terminus of N-803 [98]. N-820 activated primary NK cells to enhance ADCC and induced apoptosis of B cell NHL in vitro and in BL xenografted NSG mice [98]. N-820 also significantly enhanced the cytotoxicity of exPBNK against rituximab-sensitive and -resistant BL cells in vitro and in BL xenografted NSG mice in vivo, as compared to controls [99]. Our study and others suggest that N-820 is an attractive novel agent to be combined with NK therapy for CD20^+^ relapsed/refractory B-NHL [99].

## Future directions

Although much is now established regarding the importance of NK cell number and function in the context of cancer survival, the parameters that define the optimal NK cell with respect to phenotype, function, proliferative potential, and long-term persistence are not well defined. In vitro assays that accurately reflect in vivo conditions, tumor microenvironment, trafficking, and cross-talk with other immune cells are much needed, and fully murine models have only partially filled this gap because of significant differences between mouse and human NK cell biology. It is also important to recognize that the definition of “optimal” may vary between different cancers and with different combination therapies.

This baseline understanding of optimal will be important in defining differences—pro and con—between the various starting material/sources from which NK cells are generated, expansion methods, genetic modification methods, and cytokine adjuvants. With several new options now enabling engineering of NK cells after decades of difficulty in this area, the wide variety of targeting domains, signaling and cytokine combinations, and cell sources literally provide hundreds of potential CAR NK products that are now feasible and therefor will need to be tested in robust models for each disease.

In addition, the combinations with exogenous cytokines, engagers, immune modulators, and checkpoint inhibition will result in many new options for patients that need careful investigation. To achieve the best outcome quickly, clinical investigation will require cooperation, intelligent trial design, and implementation of multi-cohort, basket, or surrogate endpoints.

Lastly, this review has focused on the relevance of NK cells in the treatment of B cell NHL. Several antibodies have been developed for T cell NHL (e.g., alemtuzumab (anti-CD52), brentuximab (anti-CD30), and mogalizumab (anti-CXCR4)), all of which mediate at least part of their anti-lymphoma efficacy through ADCC. Thus, approaches to enhancing NK cell number and activity may also be highly relevant for T cell NHL.

## Summary

NK cell-based immunotherapy holds tremendous promise for patients with B-NHL. A robust, carefully designed, and centrally coordinated systematic investigation of the available modalities should lead to significantly improved outcomes in the near future.

## References

[1] Cairo MS, Beishuizen A (2019). Childhood, adolescent and young adult non-Hodgkin lymphoma: current perspectives. Br J Haematol..

[2] Hochberg J, Flower A, Brugieres L, Cairo MS (2018). NHL in adolescents and young adults: a unique population. Pediatr Blood Cancer..

[3] Chu Y, Gardenswartz A, Termuhlen AM, Cairo MS (2019). Advances in cellular and humoral immunotherapy—implications for the treatment of poor risk childhood, adolescent, and young adult B-cell non-Hodgkin lymphoma. Br J Haematol..

[4] Cairo MS, Sposto R, Gerrard M, Auperin A, Goldman SC, Harrison L (2012). Advanced stage, increased lactate dehydrogenase, and primary site, but not adolescent age (>/= 15 years), are associated with an increased risk of treatment failure in children and adolescents with mature B-cell non-Hodgkin's lymphoma: results of the FAB LMB 96 study. J Clin Oncol..

[5] Goldman S, Smith L, Anderson JR, Perkins S, Harrison L, Geyer MB (2013). Rituximab and FAB/LMB 96 chemotherapy in children with Stage III/IV B-cell non-Hodgkin lymphoma: a Children's Oncology Group report. Leukemia..

[6] Coiffier B, Lepage E, Briere J, Herbrecht R, Tilly H, Bouabdallah R (2002). CHOP chemotherapy plus rituximab compared with CHOP alone in elderly patients with diffuse large-B-cell lymphoma. N Engl J Med..

[7] Habermann TM, Weller EA, Morrison VA, Gascoyne RD, Cassileth PA, Cohn JB (2006). Rituximab-CHOP versus CHOP alone or with maintenance rituximab in older patients with diffuse large B-cell lymphoma. J Clin Oncol..

[8] Cairo M, Auperin A, Perkins SL, Pinkerton R, Harrison L, Goldman S (2018). Overall survival of children and adolescents with mature B cell non-Hodgkin lymphoma who had refractory or relapsed disease during or after treatment with FAB/LMB 96: a report from the FAB/LMB 96 study group. Br J Haematol..

[9] Neelapu SS, Adkins S, Ansell SM, Brody J, Cairo MS, Friedberg JW (2020). Society for Immunotherapy of Cancer (SITC) clinical practice guideline on immunotherapy for the treatment of lymphoma. J Immunother Cancer..

[10] Locke FL, Ghobadi A, Jacobson CA, Miklos DB, Lekakis LJ, Oluwole OO (2019). Long-term safety and activity of axicabtagene ciloleucel in refractory large B-cell lymphoma (ZUMA-1): a single-arm, multicentre, phase 1-2 trial. Lancet Oncol..

[11] Schuster SJ, Bishop MR, Tam CS, Waller EK, Borchmann P, McGuirk JP (2019). Tisagenlecleucel in adult relapsed or refractory diffuse large B-cell lymphoma. N Engl J Med..

[12] Wang M, Munoz J, Goy A, Locke FL, Jacobson CA, Hill BT (2020). KTE-X19 CAR T-cell therapy in relapsed or refractory mantle-cell lymphoma. N Engl J Med..

[13] Liu E, Marin D, Banerjee P, Macapinlac HA, Thompson P, Basar R (2020). Use of CAR-transduced natural killer cells in CD19-positive lymphoid tumors. N Engl J Med..

[14] Cooley S, Parham P, Miller JS (2018). Strategies to activate NK cells to prevent relapse and induce remission following hematopoietic stem cell transplantation. Blood..

[15] Lamb MG, Rangarajan HG, Tullius BP, Lee DA (2021). Natural killer cell therapy for hematologic malignancies: successes, challenges, and the future. Stem Cell Res Ther..

[16] Lee DA (2019). Cellular therapy: Adoptive immunotherapy with expanded natural killer cells. Immunol Rev..

[17] Mamo T, Williams SM, Kinney S, Tessier KM, DeFor TE, Cooley S (2021). Infusion reactions in natural killer cell immunotherapy: a retrospective review. Cytotherapy..

[18] Alvaro-Naranjo T, Lejeune M, Salvado-Usach MT, Bosch-Princep R, Reverter-Branchat G, Jaen-Martinez J (2005). Tumor-infiltrating cells as a prognostic factor in Hodgkin's lymphoma: a quantitative tissue microarray study in a large retrospective cohort of 267 patients. Leuk Lymphoma..

[19] Xu-Monette ZY, Xiao M, Au Q, Padmanabhan R, Xu B, Hoe N (2019). Immune profiling and quantitative analysis decipher the clinical role of immune-checkpoint expression in the tumor immune microenvironment of DLBCL. Cancer Immunol Res..

[20] Plonquet A, Haioun C, Jais JP, Debard AL, Salles G, Bene MC (2007). Peripheral blood natural killer cell count is associated with clinical outcome in patients with aaIPI 2-3 diffuse large B-cell lymphoma. Ann Oncol..

[21] • Bachanova V, Sarhan D, DeFor TE, Cooley S, Panoskaltsis-Mortari A, Blazar BR, et al. Haploidentical natural killer cells induce remissions in non-Hodgkin lymphoma patients with low levels of immune-suppressor cells. Cancer Immunol Immunother. 2018;67(3):483–94. This is the first publication a clinical trial demonstrating the efficacy of NK cell adoptive immunotherapy for patients with lymphoma 10.1007/s00262-017-2100-1 PMC6055922 29218366

[22] Ninomiya S, Hara T, Tsurumi H, Hoshi M, Kanemura N, Goto N (2011). Indoleamine 2,3-dioxygenase in tumor tissue indicates prognosis in patients with diffuse large B-cell lymphoma treated with R-CHOP. Ann Hematol..

[23] Chiu J, Ernst DM, Keating A (2018). Acquired natural killer cell dysfunction in the tumor microenvironment of classic Hodgkin lymphoma. Front Immunol..

[24] Vari F, Arpon D, Keane C, Hertzberg MS, Talaulikar D, Jain S (2018). Immune evasion via PD-1/PD-L1 on NK cells and monocyte/macrophages is more prominent in Hodgkin lymphoma than DLBCL. Blood..

[25] Chen X, Zang Y, Li D, Guo J, Wang Y, Lin Y (2020). IDO, TDO, and AHR overexpression is associated with poor outcome in diffuse large B-cell lymphoma patients in the rituximab era. Medicine (Baltimore)..

[26] Dong L, Lv H, Li W, Song Z, Li L, Zhou S (2016). Co-expression of PD-L1 and p-AKT is associated with poor prognosis in diffuse large B-cell lymphoma via PD-1/PD-L1 axis activating intracellular AKT/mTOR pathway in tumor cells. Oncotarget..

[27] Yang ZZ, Grote DM, Ziesmer SC, Xiu B, Yates NR, Secreto FJ (2013). Soluble and membrane-bound TGF-beta-mediated regulation of intratumoral T cell differentiation and function in B-cell non-Hodgkin lymphoma. PLoS One..

[28] Porrata LF, Inwards DJ, Micallef IN, Johnston PB, Ansell SM, Hogan WJ (2010). Interleukin-15 affects patient survival through natural killer cell recovery after autologous hematopoietic stem cell transplantation for non-Hodgkin lymphomas. Clin Dev Immunol..

[29] Hattori N, Saito B, Sasaki Y, Shimada S, Murai S, Abe M (2018). Status of natural killer cell recovery in day 21 bone marrow after allogeneic hematopoietic stem cell transplantation predicts clinical outcome. Biol Blood Marrow Transplant..

[30] Kansagra A, Inwards DJ, Ansell SM, Micallef IN, Johnston PB, Hogan WJ (2018). Infusion of autograft natural killer cell/CD14(+)HLA-DR(DIM) cell ratio predicts survival in lymphoma post autologous stem cell transplantation. Bone Marrow Transplant..

[31] Bachanova V, Weisdorf DJ, Wang T, Marsh SGE, Trachtenberg E, Haagenson MD (2016). Donor KIR B genotype improves progression-free survival of non-Hodgkin lymphoma patients receiving unrelated donor transplantation. Biol Blood Marrow Transplant..

[32] Gordan LN, Sugrue MW, Lynch JW, Williams KD, Khan SA, Moreb JS (2003). Correlation of early lymphocyte recovery and progression-free survival after autologous stem-cell transplant in patients with Hodgkin's and non-Hodgkin's Lymphoma. Bone Marrow Transplant..

[33] Raspadori D, Lauria F, Ventura MA, Tazzari PL, Ferrini S, Miggiano MC (1995). Low doses of rIL2 after autologous bone marrow transplantation induce a "prolonged" immunostimulation of NK compartment in high-grade non-Hodgkin's lymphomas. Ann Hematol..

[34] Burns LJ, Weisdorf DJ, DeFor TE, Vesole DH, Repka TL, Blazar BR (2003). IL-2-based immunotherapy after autologous transplantation for lymphoma and breast cancer induces immune activation and cytokine release: a phase I/II trial. Bone Marrow Transplant..

[35] Miller JS, Tessmer-Tuck J, Pierson BA, Weisdorf D, McGlave P, Blazar BR (1997). Low dose subcutaneous interleukin-2 after autologous transplantation generates sustained in vivo natural killer cell activity. Biol Blood Marrow Transplant..

[36] Lister J, Rybka WB, Donnenberg AD, deMagalhaes-Silverman M, Pincus SM, Bloom EJ (1995). Autologous peripheral blood stem cell transplantation and adoptive immunotherapy with activated natural killer cells in the immediate posttransplant period. Clin Cancer Res..

[37] Benyunes MC, Higuchi C, York A, Lindgren C, Thompson JA, Buckner CD (1995). Immunotherapy with interleukin 2 with or without lymphokine-activated killer cells after autologous bone marrow transplantation for malignant lymphoma: a feasibility trial. Bone Marrow Transplant..

[38] Leemhuis T, Wells S, Scheffold C, Edinger M, Negrin RS (2005). A phase I trial of autologous cytokine-induced killer cells for the treatment of relapsed Hodgkin disease and non-Hodgkin lymphoma. Biol Blood Marrow Transplant..

[39] Margolin KA, Aronson FR, Sznol M, Atkins MB, Ciobanu N, Fisher RI (1991). Phase II trial of high-dose interleukin-2 and lymphokine-activated killer cells in Hodgkin's disease and non-Hodgkin's lymphoma. J Immunother (1991)..

[40] Klingemann H, Grodman C, Cutler E, Duque M, Kadidlo D, Klein AK (2013). Autologous stem cell transplant recipients tolerate haploidentical related-donor natural killer cell-enriched infusions. Transfusion..

[41] Bachanova V, Burns LJ, McKenna DH, Curtsinger J, Panoskaltsis-Mortari A, Lindgren BR (2010). Allogeneic natural killer cells for refractory lymphoma. Cancer Immunol Immunother..

[42] Yang Y, Lim O, Kim TM, Ahn YO, Choi H, Chung H (2016). Phase I study of random healthy donor-derived allogeneic natural killer cell therapy in patients with malignant lymphoma or advanced solid tumors. Cancer Immunol Res..

[43] Tanaka J, Tanaka N, Wang YH, Mitsuhashi K, Ryuzaki M, Iizuka Y (2020). Phase I study of cellular therapy using ex vivo expanded natural killer cells from autologous peripheral blood mononuclear cells combined with rituximab-containing chemotherapy for relapsed CD20-positive malignant lymphoma patients. Haematologica..

[44] Koepsell SA, Miller JS, McKenna DH (2013). Natural killer cells: a review of manufacturing and clinical utility. Transfusion..

[45] Klanova M, Oestergaard MZ, Trneny M, Hiddemann W, Marcus R, Sehn LH (2019). Prognostic impact of natural killer cell count in follicular lymphoma and diffuse large B-cell lymphoma patients treated with immunochemotherapy. Clin Cancer Res..

[46] Ansell SM, Geyer SM, Maurer MJ, Kurtin PJ, Micallef IN, Stella P (2006). Randomized phase II study of interleukin-12 in combination with rituximab in previously treated non-Hodgkin's lymphoma patients. Clin Cancer Res..

[47] Eisenbeis CF, Grainger A, Fischer B, Baiocchi RA, Carrodeguas L, Roychowdhury S (2004). Combination immunotherapy of B-cell non-Hodgkin's lymphoma with rituximab and interleukin-2: a preclinical and phase I study. Clin Cancer Res..

[48] Holmberg LA, Maloney D, Bensinger W (2006). Immunotherapy with rituximab/interleukin-2 after autologous stem cell transplantation as treatment for CD20+ non-Hodgkin's lymphoma. Clin Lymphoma Myeloma..

[49] Robertson MJ, Stamatkin CW, Pelloso D, Weisenbach J, Prasad NK, Safa AR (2018). A dose-escalation study of recombinant human interleukin-18 in combination with ofatumumab after autologous peripheral blood stem cell transplantation for lymphoma. J Immunother..

[50] Gluck WL, Hurst D, Yuen A, Levine AM, Dayton MA, Gockerman JP (2004). Phase I studies of interleukin (IL)-2 and rituximab in B-cell non-hodgkin's lymphoma: IL-2 mediated natural killer cell expansion correlations with clinical response. Clin Cancer Res..

[51] Lopez-Diaz de Cerio A, Garcia-Munoz R, Pena E, Panizo A, Feliu J, Giraldo P (2020). Maintenance therapy with ex vivo expanded lymphokine-activated killer cells and rituximab in patients with follicular lymphoma is safe and may delay disease progression. Br J Haematol..

[52] Romee R, Cooley S, Berrien-Elliott MM, Westervelt P, Verneris MR, Wagner JE (2018). First-in-human phase 1 clinical study of the IL-15 superagonist complex ALT-803 to treat relapse after transplantation. Blood..

[53] Foltz JA, Hess BT, Bachanova V, Bartlett NL, Berrien-Elliott MM, McClain E (2021). Phase 1 trial of N-803, an IL-15 receptor agonist, with rituximab in patients with indolent non-Hodgkin lymphoma. Clin Cancer Res..

[54] Barth MJ, Chu Y, Hanley PJ, Cairo MS (2016). Immunotherapeutic approaches for the treatment of childhood, adolescent and young adult non-Hodgkin lymphoma. Br J Haematol..

[55] Nayyar G, Chu Y, Cairo MS (2019). Overcoming resistance to natural killer cell based immunotherapies for solid tumors. Front Oncol..

[56] Liu E, Tong Y, Dotti G, Shaim H, Savoldo B, Mukherjee M (2018). Cord blood NK cells engineered to express IL-15 and a CD19-targeted CAR show long-term persistence and potent antitumor activity. Leukemia..

[57] Miller JS, Soignier Y, Panoskaltsis-Mortari A, McNearney SA, Yun GH, Fautsch SK (2005). Successful adoptive transfer and in vivo expansion of human haploidentical NK cells in patients with cancer. Blood..

[58] Gunesch JT, Angelo LS, Mahapatra S, Deering RP, Kowalko JE, Sleiman P (2019). Genome-wide analyses and functional profiling of human NK cell lines. Mol Immunol..

[59] Gong JH, Maki G, Klingemann HG (1994). Characterization of a human cell line (NK-92) with phenotypical and functional characteristics of activated natural killer cells. Leukemia..

[60] Boissel L, Klingemann H, Campbell K, Nichols K, Toneguzzo F, Marcus P, et al. An ‘off the shelf,’ GMP-grade, IL-2-independent NK cell line expressing the high-affinity Fc-receptor to augment antibody therapeutics. Cancer Res. 2016;76:2302–2.

[61] Mehta RS, Shpall EJ, Rezvani K (2015). Cord blood as a source of natural killer cells. Front Med (Lausanne)..

[62] Vasu S, Berg M, Davidson-Moncada J, Tian X, Cullis H, Childs RW. A novel method to expand large numbers of CD56(+) natural killer cells from a minute fraction of selectively accessed cryopreserved cord blood for immunotherapy after transplantation. Cytotherapy. 2015;17(11):1582–93. 10.1016/j.jcyt.2015.07.020 PMC7241296 26432560

[63] Shah N, Martin-Antonio B, Yang H, Ku S, Lee DA, Cooper LJ (2013). Antigen presenting cell-mediated expansion of human umbilical cord blood yields log-scale expansion of natural killer cells with anti-myeloma activity. PloS one..

[64] Ayello J, van de Ven C, Cairo E, Hochberg J, Baxi L, Satwani P (2009). Characterization of natural killer and natural killer-like T cells derived from ex vivo expanded and activated cord blood mononuclear cells: implications for adoptive cellular immunotherapy. Exp Hematol..

[65] Cany J, van der Waart AB, Spanholtz J, Tordoir M, Jansen JH, van der Voort R (2015). Combined IL-15 and IL-12 drives the generation of CD34(+)-derived natural killer cells with superior maturation and alloreactivity potential following adoptive transfer. Oncoimmunology..

[66] Spanholtz J, Tordoir M, Eissens D, Preijers F, van der Meer A, Joosten I (2010). High log-scale expansion of functional human natural killer cells from umbilical cord blood CD34-positive cells for adoptive cancer immunotherapy. PLoS One..

[67] Woll PS, Martin CH, Miller JS, Kaufman DS (2005). Human embryonic stem cell-derived NK cells acquire functional receptors and cytolytic activity. J Immunol..

[68] Zhu H, Lai YS, Li Y, Blum RH, Kaufman DS (2018). Concise review: human pluripotent stem cells to produce cell-based cancer immunotherapy. Stem Cells..

[69] Yamamoto K, Blum R, Kaufman DS (2020). ADAM17-deficient pluripotent stem cell-derived natural killer cells possess improved antibody-dependent cellular cytotoxicity and antitumor activity. Blood..

[70] Kundu S, Gurney M, O'Dwyer M (2021). Generating natural killer cells for adoptive transfer: expanding horizons. Cytotherapy..

[71] Ahn YO, Kim S, Kim TM, Song EY, Park MH, Heo DS (2013). Irradiated and activated autologous PBMCs induce expansion of highly cytotoxic human NK cells in vitro. J Immunother..

[72] Lim O, Lee Y, Chung H, Her JH, Kang SM, Jung MY (2013). GMP-compliant, large-scale expanded allogeneic natural killer cells have potent cytolytic activity against cancer cells in vitro and in vivo. PLoS One..

[73] Delso-Vallejo M, Kollet J, Koehl U, Huppert V (2017). Influence of irradiated peripheral blood mononuclear cells on both ex vivo proliferation of human natural killer cells and change in cellular property. Front Immunol..

[74] Berg M, Lundqvist A, McCoy P, Samsel L, Fan Y, Tawab A (2009). Clinical-grade ex vivo-expanded human natural killer cells up-regulate activating receptors and death receptor ligands and have enhanced cytolytic activity against tumor cells. Cytotherapy..

[75] Imai C, Iwamoto S, Campana D (2005). Genetic modification of primary natural killer cells overcomes inhibitory signals and induces specific killing of leukemic cells. Blood..

[76] Ayello J, Hochberg J, Flower A, Chu Y, Baxi LV, Quish W (2017). Genetically re-engineered K562 cells significantly expand and functionally activate cord blood natural killer cells: Potential for adoptive cellular immunotherapy. Exp Hematol..

[77] Baek HJ, Kim JS, Yoon M, Lee JJ, Shin MG, Ryang DW (2013). Ex vivo expansion of natural killer cells using cryopreserved irradiated feeder cells. Anticancer Res..

[78] Denman CJ, Senyukov VV, Somanchi SS, Phatarpekar PV, Kopp LM, Johnson JL (2012). Membrane-bound IL-21 promotes sustained ex vivo proliferation of human natural killer cells. PloS one..

[79] Frei GM, Persi N, Lador C, Peled A, Cohen YC, Nagler A (2011). Nicotinamide, a form of vitamin B3, promotes expansion of natural killer cells that display increased in vivo survival and cytotoxic activity. Blood..

[80] Oyer JL, Igarashi RY, Kulikowski AR, Colosimo DA, Solh MM, Zakari A (2015). Generation of highly cytotoxic natural killer cells for treatment of acute myelogenous leukemia using a feeder-free, particle-based approach. Biol Blood Marrow Transplant..

[81] Oyer JL, Pandey V, Igarashi RY, Somanchi SS, Zakari A, Solh M (2016). Natural killer cells stimulated with PM21 particles expand and biodistribute in vivo: Clinical implications for cancer treatment. Cytotherapy..

[82] Naeimi Kararoudi M, Tullius BP, Chakravarti N, Pomeroy EJ, Moriarity BS, Beland K (2020). Genetic and epigenetic modification of human primary NK cells for enhanced antitumor activity. Semin Hematol..

[83] Shimasaki N, Fujisaki H, Cho D, Masselli M, Lockey T, Eldridge P (2012). A clinically adaptable method to enhance the cytotoxicity of natural killer cells against B-cell malignancies. Cytotherapy..

[84] Chu Y, Hochberg J, Yahr A, Ayello J, van de Ven C, Barth M (2015). Targeting CD20+ aggressive B-cell non-Hodgkin lymphoma by anti-CD20 CAR mRNA-modified expanded natural killer cells in vitro and in NSG mice. Cancer Immunol Res..

[85] Chu Y, Yahr A, Huang B, Ayello J, Barth M, Cairo MS (2017). Romidepsin alone or in combination with anti-CD20 chimeric antigen receptor expanded natural killer cells targeting Burkitt lymphoma in vitro and in immunodeficient mice. Oncoimmunology..

[86] Ravi D, Sarkar S, Purvey S, Passero F, Beheshti A, Chen Y (2020). Interaction kinetics with transcriptomic and secretory responses of CD19-CAR natural killer-cell therapy in CD20 resistant non-hodgkin lymphoma. Leukemia..

[87] Romee R, Schneider SE, Leong JW, Chase JM, Keppel CR, Sullivan RP (2012). Cytokine activation induces human memory-like NK cells. Blood..

[88] Gang M, Wong P, Berrien-Elliott MM, Fehniger TA (2020). Memory-like natural killer cells for cancer immunotherapy. Semin Hematol..

[89] Ni J, Miller M, Stojanovic A, Garbi N, Cerwenka A (2012). Sustained effector function of IL-12/15/18-preactivated NK cells against established tumors. J Exp Med..

[90] Wagner JA, Berrien-Elliott MM, Rosario M, Leong JW, Jewell BA, Schappe T (2017). Cytokine-induced memory-like differentiation enhances unlicensed natural killer cell antileukemia and FcgammaRIIIa-triggered responses. Biol Blood Marrow Transplant..

[91] Gang M, Marin ND, Wong P, Neal CC, Marsala L, Foster M (2020). CAR-modified memory-like NK cells exhibit potent responses to NK-resistant lymphomas. Blood..

[92] Goodridge JP, Mahmood S, Zhu H, Gaidarova S, Blum R, Bjordahl R (2019). FT596: Translation of first-of-kind multi-antigen targeted off-the-shelf CAR-NK cell with engineered persistence for the treatment of B cell malignancies. Blood..

[93] Awasthi A, Ayello J, Van de Ven C, Elmacken M, Sabulski A, Barth MJ (2015). Obinutuzumab (GA101) compared to rituximab significantly enhances cell death and antibody-dependent cytotoxicity and improves overall survival against CD20(+) rituximab-sensitive/-resistant Burkitt lymphoma (BL) and precursor B-acute lymphoblastic leukaemia (pre-B-ALL): potential targeted therapy in patients with poor risk CD20(+) BL and pre-B-ALL. Br J Haematol..

[94] Gleason MK, Verneris MR, Todhunter DA, Zhang B, McCullar V, Zhou SX (2012). Bispecific and trispecific killer cell engagers directly activate human NK cells through CD16 signaling and induce cytotoxicity and cytokine production. Mol Cancer Ther..

[95] Felices M, Kodal B, Hinderlie P, Kaminski MF, Cooley S, Weisdorf DJ (2019). Novel CD19-targeted TriKE restores NK cell function and proliferative capacity in CLL. Blood Adv..

[96] Reusch U, Burkhardt C, Fucek I, Le Gall F, Le Gall M, Hoffmann K (2014). A novel tetravalent bispecific TandAb (CD30/CD16A) efficiently recruits NK cells for the lysis of CD30+ tumor cells. MAbs..

[97] Rosario M, Liu B, Kong L, Collins LI, Schneider SE, Chen X (2016). The IL-15-Based ALT-803 complex enhances FcgammaRIIIa-triggered NK cell responses and in vivo clearance of B cell lymphomas. Clin Cancer Res..

[98] Liu B, Kong L, Han K, Hong H, Marcus WD, Chen X (2016). A novel fusion of ALT-803 (interleukin (IL)-15 superagonist) with an antibody demonstrates antigen-specific antitumor responses. J Biol Chem..

[99] Chu Y, Nayyar G, Kham SN, Rosenblum JM, Soon-Shiong P, Lee J (2020). Novel cytokine-antibody fusion protein, N-820, to enhance the functions of ex vivo expanded natural killer cells against Burkitt lymphoma. J Immunother Cancer..

[100] Mossner E, Brunker P, Moser S, Puntener U, Schmidt C, Herter S (2010). Increasing the efficacy of CD20 antibody therapy through the engineering of a new type II anti-CD20 antibody with enhanced direct and immune effector cell-mediated B-cell cytotoxicity. Blood..

[101] Marcus R, Davies A, Ando K, Klapper W, Opat S, Owen C (2017). Obinutuzumab for the First-Line Treatment of Follicular Lymphoma. N Engl J Med..

[102] Felices M, Lenvik TR, Davis ZB, Miller JS, Vallera DA (2016). Generation of BiKEs and TriKEs to improve NK cell-mediated targeting of tumor cells. Methods Mol Biol..

[103] Steel JC, Waldmann TA, Morris JC (2012). Interleukin-15 biology and its therapeutic implications in cancer. Trends Pharmacol Sci..

[104] Han KP, Zhu X, Liu B, Jeng E, Kong L, Yovandich JL (2011). IL-15:IL-15 receptor alpha superagonist complex: high-level co-expression in recombinant mammalian cells, purification and characterization. Cytokine..

[105] Zhu X, Marcus WD, Xu W, Lee HI, Han K, Egan JO (2009). Novel human interleukin-15 agonists. J Immunol..

